# Re-engineering Plant Phenylpropanoid Metabolism With the Aid of Synthetic Biosensors

**DOI:** 10.3389/fpls.2021.701385

**Published:** 2021-09-16

**Authors:** Savio S. Ferreira, Mauricio S. Antunes

**Affiliations:** Department of Biological Sciences and BioDiscovery Institute, University of North Texas, Denton, TX, United States

**Keywords:** phenylpropanoids, metabolic engineering, synthetic biology, biosensors, genetic circuits

## Abstract

Phenylpropanoids comprise a large class of specialized plant metabolites with many important applications, including pharmaceuticals, food nutrients, colorants, fragrances, and biofuels. Therefore, much effort has been devoted to manipulating their biosynthesis to produce high yields in a more controlled manner in microbial and plant systems. However, current strategies are prone to significant adverse effects due to pathway complexity, metabolic burden, and metabolite bioactivity, which still hinder the development of tailor-made phenylpropanoid biofactories. This gap could be addressed by the use of biosensors, which are molecular devices capable of sensing specific metabolites and triggering a desired response, as a way to sense the pathway’s metabolic status and dynamically regulate its flux based on specific signals. Here, we provide a brief overview of current research on synthetic biology and metabolic engineering approaches to control phenylpropanoid synthesis and phenylpropanoid-related biosensors, advocating for the use of biosensors and genetic circuits as a step forward in plant synthetic biology to develop autonomously-controlled phenylpropanoid-producing plant biofactories.

## Introduction

**Figure 1:**
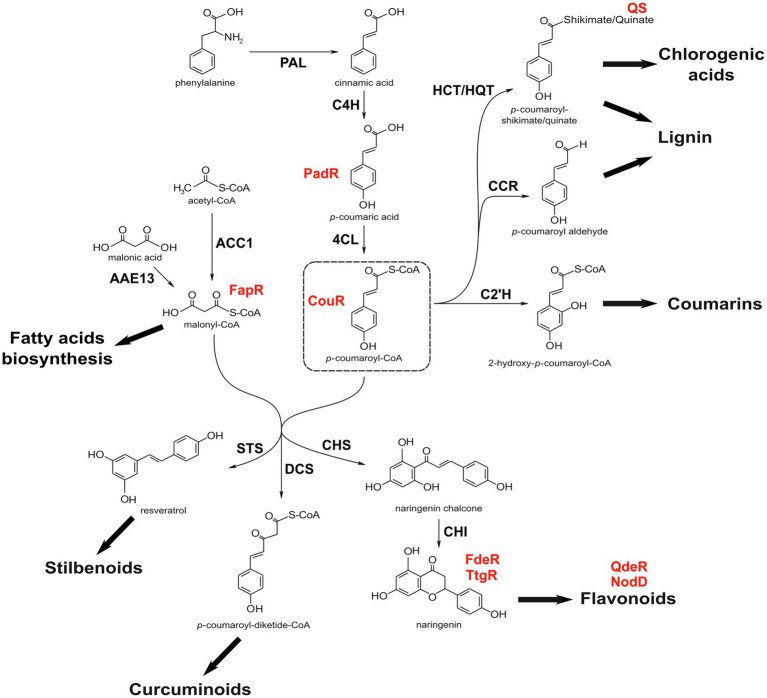
Simplified phenylpropanoid pathway. Figure shows the conversion of phenylalanine into *p*-coumaroyl-CoA, from where most phenylpropanoid branches derive. Some reactions are not depicted for simplification and several phenylpropanoid derivates can have different branching points in addition to the ones shown here. Some of the sensor proteins discussed in the text are shown in red, right next to its respective ligand. Note that QS binds to quinic acid free form (quinate), not to *p*-coumaroyl-quinate. PAL, phenylalanine ammonia lyase; C4H, cinnamate 4-hydroxylase; 4CL, 4-coumarate-CoA ligase; HCT, *p*-hydroxycinnamoyl-CoA:shikimate *p*-hydroxycinnamoyl transferase; HQT, hydroxycinnamoyl-CoA:quinate hydroxycinnamoyl transferase; CCR, cinnamoyl-CoA reductase; C2’H, *p*-coumaroyl CoA 2'-hydroxylase; ACC1, acetyl-CoA carboxylase; AAE13, malonyl-CoA synthetase; STS, stilbene synthase; DCS, diketide-CoA synthase; CHS, chalcone synthase; and CHI, chalcone isomerase.

Phenylpropanoid metabolism is a major anabolic pathway in plants, with several branches involved in the synthesis of a variety of specialized metabolites (Figure 1), such as lignin, phenolic acids, curcuminoids, coumarins, stilbenes, and the large group of flavonoids (anthocyanins, flavonols, flavones, among others). As its diversity suggests, this pathway is tightly and dynamically regulated to allow the plant to direct carbon flux into each branch as needed (Xiao et al., 2018; Ma and Constabel, 2019; Nabavi et al., 2020). The initial steps of the phenylpropanoid pathway convert the amino acid phenylalanine into *p*-coumaroyl-CoA by the sequential action of phenylalanine ammonia lyase (PAL), cinnamate 4-hydroxylase (C4H), and 4-coumarate-CoA ligase (4CL) enzymes (Figure 1). From that point, carbon flux is diverted to almost all other branches and the resulting metabolites have a plethora of physiological roles, such as regulators of growth and development, stress tolerance, and microbial symbiosis (Bieza and Lois, 2001; Dixon et al., 2002; Mandal et al., 2010). This wide range of functions can be channeled for industrial purposes, like pharmaceuticals, colorants, fragrances, and food nutrients (Chen et al., 2020), which has prompted synthetic biologists to investigate strategies to produce these metabolites in a more efficient and controlled manner.

Efforts to manipulate phenylpropanoid metabolism have consisted mostly of heterologous reconstitution of pathway branches in bacteria or yeast hosts (Pyne et al., 2019; Chen et al., 2020; Marsafari et al., 2020). Initial approaches introduced plant enzymes into microbial chassis to overproduce a phenylpropanoid-derived metabolite. However, significant adverse effects pose limitations to this strategy, such as growth impairment due to carbon flux diverted to the heterologous pathway instead of the host’s primary metabolism, or accumulation of toxic products (Gong et al., 2017; Li et al., 2018). These issues have led to the development of more complex synthetic genetic circuits that are capable of dynamically controlling target metabolite biosynthesis, as well as efforts to evolve strains with higher tolerance (Gong et al., 2017; Chen et al., 2020). In this context, biosensors have emerged as a valuable tool, as they can be designed to sense key pathway compounds and trigger a desired response. Accordingly, recent studies have used biosensors to improve phenylpropanoid production in bacteria and yeast (Pyne et al., 2019; Marsafari et al., 2020).

## Plant Biofactories

Despite the advantages of microbial systems, such as being more amenable to genetic manipulation and controlled growth conditions, some particularities of plants advocate in their favor to be used as chassis for designing biofactories (Maeda, 2019; Molina-Hidalgo et al., 2020). Plants can accumulate larger amounts of biomass, which can be directly correlated to the desired product yield. In the case of phenylpropanoids, plants can accumulate large quantities of these metabolites (e.g., in vacuoles), whereas this may be a limiting factor in bacteria, as many phenylpropanoids have antimicrobial activity. Moreover, the enormous natural diversity of plant genes and pathways that lead to unique specialized metabolites, which can be species- or environment-specific, can be better harnessed by using plant chassis. Plants are natural producers of phenylpropanoids; therefore, there is no need to reconstitute the whole pathway heterologously. It would be quite challenging, if not impossible, to design a microbial host comprising all enzymes required to mimic the phenylpropanoid diversity found in plants. In addition, plant enzymes may not function as efficiently in microbial hosts as in plants, especially in prokaryotes lacking post-translational modifications and organelles for enzyme compartmentalization. Finally, plants can be readily edible or require minimal processing, and some products (e.g., pharmaceuticals) can take advantage of this delivery method, greatly reducing costs associated with the production chain. For example, oral delivery of *Artemisia annua* dried leaves is an effective alternative to treat malaria, bypassing the expensive purification step of the active compound, artemisinin (Daddy et al., 2017). Similarly, heterologous expression of immunogenic virus-like particles in plants have been studied as edible vaccines for diseases like dengue, tuberculosis, and rabies (He et al., 2021). Nevertheless, manipulation of plant metabolism using synthetic biology approaches still faces major challenges, such as low yields, pleiotropic effects, and therefore, better strategies for precise control of pathways must be developed. In this case, biosensors stand out as a promising device to allow implementation of autonomous and dynamic control of phenylpropanoid biosynthesis in plants.

## Plant Phenylpropanoid Engineering

Most efforts to engineer phenylpropanoid synthesis in plants have targeted the constitutive up or downregulation of key genes (transcriptional regulators or enzymes) within the pathway, or in competing or precursor-producing pathways, to change the content of a desired metabolite. Significant progress has been made by targeting transcription factors (TFs) that regulate enzymes involved in phenylpropanoid biosynthesis. For example, fruit-specific expression of the *Arabidopsis* AtMYB12 TF in tomato increased carbon flux through the shikimate and aromatic amino acid biosynthesis pathways, which in turn fueled the phenylpropanoid pathway, leading to higher chlorogenic acid and flavonol contents in these fruits (Zhang et al., 2015). Moreover, constitutive overexpression of AtMYB12 (Misra et al., 2010) in tobacco increased insect resistance due to higher flavonol accumulation. Similarly, enzymes have also been successfully targeted; for instance, CRISPR-mediated mutations in soybean flavanone 3'-hydroxylase genes promoted accumulation of isoflavones in leaves, which led to resistance against the soybean mosaic virus (Zhang et al., 2020); CRISPR-mediated knock-out of the same gene in petunia plants produced a pale purplish pink flower color phenotype, instead of the wild type purple violet (Yu et al., 2020).

The use of plant biomass as feedstock for the production of biofuels and biomaterials has been a major research topic in the past two decades, pushed forward mainly by environmental concerns related to non-renewable materials. However, plant cell walls have evolved to be resistant to degradation, imposing a major bottleneck for their use as an energy source, and lignin is one of the main factors responsible for this recalcitrance (Himmel et al., 2007). Thus, numerous studies have focused on reducing lignin content, altering its structure, or its composition, to reduce biomass recalcitrance. Downregulation or knock-out of key genes involved in the biosynthesis of lignin monomers (monolignols) have been the preferred approaches (Chanoca et al., 2019; Halpin, 2019; Mahon and Mansfield, 2019). Nonetheless, more precise strategies have also been tested successfully, such as use of vessel-specific promoters to restrict lignification to where it is essential (Yang et al., 2013; De Meester et al., 2018), and altering lignin structure or monolignol composition (Mottiar et al., 2016; Mahon and Mansfield, 2019; Ralph et al., 2019).

Phenylpropanoid pathway perturbations, especially the vast majority that use constitutive promoters, are prone to result in epistatic effects, significantly altering the plant’s metabolome, transcriptome, and hence, its physiology (Vanholme et al., 2012, 2019; Halpin, 2019). For example, flavonol-enriched, AtMYB12-expressing tomato plants showed decreased total sugar content (as carbon flux was diverted from primary metabolism), which may affect tomato taste (Zhang et al., 2015). Reduced lignin content tends to affect plant growth and biomass (Ha et al., 2021) and, even with the use of remedial strategies to address the dwarf phenotype, effects on metabolome and plant responses to pathogens have still been reported (Halpin, 2019; Muro-Villanueva et al., 2019; Ha et al., 2021). Moreover, some phenylpropanoid intermediates can have bioactive properties and affect plant physiology, such as ferulic acid, which might affect cell proliferation (Xue et al., 2015), and cis-cinnamic acid, a light-isomerization product of trans-cinnamic acid that inhibits auxin efflux (El Houari et al., 2021). Thus, alterations in phenolic profile caused by constitutive expression or permanent mutations can lead to unforeseen effects. The use of autoregulatory synthetic genetic circuits controlled by biosensors can address these issues by turning transgene expression on and off as needed to reduce the metabolic burden, i.e., the energy demand for transgene expression and for keeping cells’ homeostasis given the metabolic perturbations caused by the transgene(s). These biosensors may help the organism sense its metabolic status and trigger a response accordingly, greatly improving control of the output, thereby potentially minimizing adverse effects.

## Biosensors as Metabolite-Responsive On–Off Switches

Biosensors are molecular devices that can be a protein or an RNA molecule, which bind to a specific metabolite (also called ligand or inducer) and convert the changes in intracellular metabolite concentration into an output. Transcription factor sensor proteins are widely found in bacteria, and belong to different protein families, such as MarR, TetR, and LysR (Fernandez-López et al., 2015; Deochand and Grove, 2017); these proteins possess a sequence specific DNA-binding domain (DBD) and a ligand-binding domain (LBD). They naturally regulate the expression of operons responsible for metabolizing the sensed molecule, e.g., antibiotics and carbon sources (Fernandez-López et al., 2015). Generally, these proteins act as transcriptional repressors by binding to an operator sequence (also known as response element) in the promoter of target genes, preventing their expression in the absence of the ligand; interaction with the ligand causes conformational changes in the DBD that lead to dissociation of the repressor from the DNA, allowing RNA polymerase to initiate transcription. Some biosensors can function as inducers by binding to the operator sequence only after interacting with the ligand, which will ultimately aid in recruiting the transcriptional machinery to induce gene expression (Hossain et al., 2020). In a few known cases, the same biosensor can act as both repressor and activator (Xu et al., 2014; De Paepe et al., 2019). Small cis-regulatory RNA elements called riboswitches are another class of biosensors, in which ligand binding to a riboswitch LBD (aptamer) stabilizes RNA secondary structures that can affect transcription or translation (Hossain et al., 2020). A third class of biosensors is based on enzymatic activities, such as membrane receptor-kinase pairs that initiate phosphorylation cascades that result in transcription of output genes (Antunes et al., 2011), and a protease-based biosensor whose input triggers target protein degradation without affecting gene expression (Agrawal et al., 2020).

**Figure 2:**
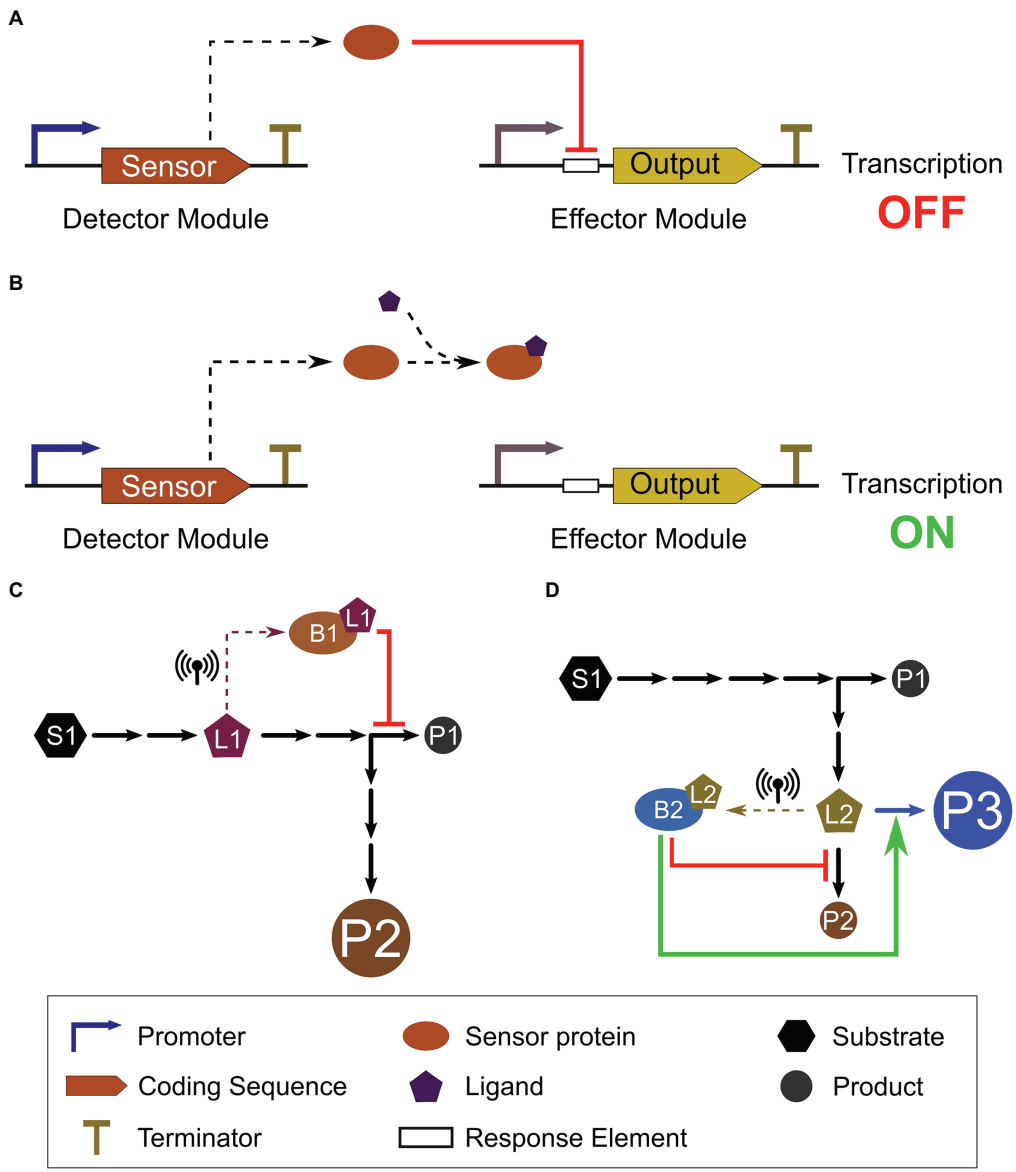
Metabolite-induced transcriptional de-repression in a biosensor system and genetic circuit application to control metabolism. **(A)** In the absence of the ligand (metabolite), the constitutively expressed repressor sensor protein binds to its response element on the target promoter (effector module), repressing expression of the output. **(B)** Conformational changes in the repressor sensor protein caused by its binding to the ligand precludes its association to the response element on the target promoter; therefore, repression is relieved and transcription of the output can occur. **(C)** A putative metabolic pathway converts substrate S1 into product P1, with a branching point to produce an alternative product P2. As the intermediate metabolite (ligand) L1 reaches a threshold concentration, it is sensed by biosensor B1 (dashed arrow), triggering repression of P1 production, thus diverting carbon flux toward P2 production and increasing P2 yield (represented by larger circle). **(D)** The same pathway can be engineered into multipart operations, for example, to produce a new product, P3, by a transgene enzyme (blue arrow). Biosensor B2 can sense the flux into P2 by the intermediate metabolite L2 (dashed arrow). As L2 reaches a threshold concentration, B2 represses production of P2, while activating the transgene expression (blue arrow), yielding the new product P3. These biosensors can act directly or indirectly, depending on their mode of action (repressor or activator) and the complexity of the genetic circuit. Different inputs can be combined and layered to build more complex operations and genetic circuits. As L1 and L2 concentrations return to levels below the biosensor threshold, the circuits are deactivated, restoring pathway fluxes.

Natural sensor proteins can be used to design genetic devices that respond to ligand concentration in heterologous systems. These synthetic biosensors are generally composed of a detector module, containing the sensor protein, and an effector module, which produces an output whose expression is regulated by the sensor protein. An example of a basic biosensor device (Figures 2A,B) consists of a fluorescent protein (effector module) whose expression is transcriptionally controlled by a constitutively expressed sensor protein (detector module). As the ligand starts to accumulate in the cell, the sensor protein de-represses (or activate) the effector module and fluorescence (output) can be detected as an indirect measure of the ligand concentration. These biosensors have been used for four main purposes in microbial synthetic biology: real-time monitoring, high-throughput screening, adaptive laboratory evolution (ALE), and dynamic pathway control. For example, a flavonoid biosensor with a fluorescent output can be used to monitor the flavonoid production in real time or in a high-throughput screening to select the highest producing strains by fluorescence-activated cell sorting (Siedler et al., 2014). Coupling this screening with rounds of selection, or linking the output to provide an adaptive advantage under selective conditions can be used for ALE, improving the yield of a desired product (Mahr et al., 2015; Siedler et al., 2017). Lastly, dynamic pathway control can be used to adjust activity of a pathway by using other TFs or key enzymes in the pathway as output of the effector module (Xu et al., 2014). For instance, dynamic control of a pathway can be achieved by upregulating limiting steps only when key metabolites or specific environmental cues are detected by the biosensor and, conversely, returning to the repressed state when these signals are no longer present. Thus, dynamic control can reduce adverse effects, such as accumulation of toxic intermediates or increased metabolic burden, and maximize yield without penalties in fitness or growth.

On the other hand, intrinsic characteristics of sensor proteins or RNAs and their heterologous hosts can impose some bottlenecks to their use as a biosensor system, requiring additional steps to fine-tune their functionality. For example, the range of detectable ligand concentration (operational range) for a biosensor in the new host (e.g., plant cell) may differ from its natural microbial cell; ligand compartmentalization (e.g., in plastids) or metabolite channeling in the host may preclude ligand-biosensor interaction. Moreover, natural sensor proteins often show affinity to several structurally related molecules, which can hamper a strong ligand-dependent specific output signal, a requirement for digital-like (on–off) responses. Similarly, the correlation between ligand concentration and signal output (dynamic range) can vary significantly based on target promoter strength, type of output signal (i.e., fluorescence vs. transcription factor) and leaky expression (expression in the absence of the ligand). Low dynamic range and leaky expression could lead to lower output responses than required for an effective sensing system, or absence of a complete “off-state” respectively. Still, different approaches can be applied to address these issues, such as computer-aided protein design, statistical modeling, random or directed mutagenesis, and the assembly of chimeric biosensors and/or promoters.

## Phenylpropanoid Biosensors

Phenylpropanoid biosensors research, to our best knowledge, has been limited to microbial systems (Alvarez-Gonzalez and Dixon, 2019; Chen et al., 2020; Marsafari et al., 2020) with several phenylpropanoid-related TF sensor proteins characterized in prokaryotes (Table 1). A few of these have been engineered to be used in high-throughput screening and ALE to develop bacterial or yeast biofactory strains (Alvarez-Gonzalez and Dixon, 2019; Chen et al., 2020; Marsafari et al., 2020), especially for flavonoids. The naringenin-responsive transcription activator FdeR (*Herbaspirillum seropedicae*) and the quercetin- and kaempferol-responsive transcription repressor QdoR (*Bacillus subtilis*) were engineered to yield fluorescent output (GFP and CFP) to screen flavonoid production in *Escherichia coli* cells expressing flavonol synthase from *Arabidopsis thaliana* (Siedler et al., 2014). A 7-fold activation in fluorescent signal was detected between off- (no flavonoid) and on- (with flavonoid) states; however, saturation of the fluorescence signal was achieved with a lower ligand concentration (0.3mM) than previously reported for naringenin producing strains (~1.7mM; Xu et al., 2011), which may limit its use for screening highly productive strains. In contrast, such a saturation limit may not be an issue for their use as regulators of dynamic control in plant systems, as long as the threshold for triggering on–off switch falls inside the operational range. A FdeR-based biosensor was also built to screen naringenin-producing *Saccharomyces cerevisiae* strains (Wang et al., 2019) and its dynamic range and sensitivity were improved by adding a nuclear localization signal, demonstrating its transferability to eukaryotic cells. As most other sensor proteins, FdeR can bind to structurally similar molecules, in this case, luteolin and apigenin. To overcome this limitation, De Paepe et al. (2019) engineered chimeric versions of FdeR with another flavonoid sensor protein, NodD (*Sinorhizobium meliloti*), resulting in shifted specificity toward luteolin, and indicating that specificity can be customized to some extent. Furthermore, another naringenin biosensor, ttgR (*Pseudomonas putida*), has been optimized in *E. coli* by a combination of random and rational mutations and directed evolution to improve dynamic range and sensitivity (Meyer et al., 2019).

**Table 1:** Phenylpropanoid-related bacterial sensor proteins.

Sensor protein	Species	Ligand	Reference
HcaR	*Acinetobacter* sp.	Hydroxycinnamoyl-CoA	Parke and Ornston, 2003
FerC	*Sphingobium* sp.	Feruloyl-CoA	Kasai et al., 2012
CouR	*Rhodococcus jostii*	*p*-coumaroyl-CoA	Otani et al., 2016
PadR	*Bacillus subtilis*	*p*-coumaric acid	Siedler et al., 2017
FapR	*Bacillus subtilis*	Malonyl-CoA	Xu et al., 2014
FdeR	*Herbaspirillum seropedicae*	Naringenin	Wassem et al., 2017
TtgR	*Pseudomonas putida*	Naringenin	Meyer et al., 2019
KaeR	*Lactobacillus brevis*	Kaempferol	Pande et al., 2011
QdoR	*Bacillus subtilis*	Quercetin and kaempferol	Siedler et al., 2014
YetL	*Bacillus subtilis*	Kaempferol and apigenin	Hirooka et al., 2009
EmrR	*Escherichia coli*	Vanillin and syringaldehyde	Ho et al., 2018
VanR	*Corynebacterium glutamicum*	Vanillate	Heravi et al., 2015
DesR	*Sphingobium* sp.	Vanillate and syringate	Araki et al., 2019
YqhC	*Escherichia coli*	Vanillin	Frazão et al., 2018
PcaV	*Streptomyces coelicolor*	Vanillin and protocatechuate	Machado et al., 2019

Biosensors are restricted to detecting intracellular ligand concentration, which may be a limitation when screening cells that secrete the desired product. To overcome this, Siedler et al. (2017) engineered a *p*-coumaric acid biosensor (PadR, *B. subtilis*) into *E. coli* cells, which are permeable to this metabolite, and thus, intra and extracellular concentrations should be comparable. Next, these authors encapsulated the *E. coli* biosensor cells with *p*-coumaric acid producing yeast strains in picoliter droplets within a microfluidic device. After a few rounds of ALE with fluorescence sorting of cells, they generated a larger library of *p*-coumaric acid-producing yeast strains with increased yield.

Phenylpropanoid biosensors have also been used in screening efforts toward valorization of the cell wall polymer lignin in plant biomass. Specific enzymatic activities related to lignin degradation are of interest, as degradation products can be used as substrate to produce value-added chemicals, such as vanillin and syringaldehyde. A biosensor based on FerC (*Sphingobium* sp. SYK-6), a feruloyl-CoA sensor protein, was developed to assess lignin degradation rate from different biomass and enzyme sources, with GFP expression triggered by the biosensor with increasing concentration of the lignin degradation product feruloyl-CoA (Machado and Dixon, 2016). Similarly, a vanillin and syringaldehyde sensor protein, EmrR (*E. coli*) was used to screen a library of metagenomic fosmids to asses natural diversity of lignin degrading genes, and 147 clones were found to be able to degrade lignin into vanillin and syringaldehyde (Ho et al., 2018). Although successful, these approaches are still limited by the detection of structurally related metabolites, and they might miss other relevant enzymatic activities that lead to degradation products undetected by these biosensors.

Perhaps the only phenylpropanoid-related biosensor tested in plants, the Q-system derived from the fungus *Neurospora crassa* was adapted in *Nicotiana benthamiana* and soybean (Reis et al., 2018; Persad et al., 2020). The Q-system consists of a transcriptional activator (QF) and its repressor (QS); the ligand, quinic acid, binds to QS to relieve the repression. Quinic acid can be conjugated with trans-cinnamic acids to yield chlorogenic acids (Figure 1), a phenylpropanoid-derived molecule involved in plant defense against pathogens and UV radiation, whose antioxidant activity is of great interest to the food industry (Volpi e Silva et al., 2019). Although, QF-driven gene expression and its repression by QS have been demonstrated in plants, quinic acid-driven de-repression was not tested. Yet, this has been demonstrated in other heterologous systems, such as drosophila (Riabinina et al., 2015) and *Caenorhabditis elegans* (Wei et al., 2012), and thus quinic acid-dependent de-repression might be possible in plants as well. As quinic acid is an essential metabolite for redirecting phenylpropanoids toward chlorogenic acids, Q-system can be a key biosensor to regulate pathway branching points.

Malonyl-CoA is a precursor for fatty acid (FA) biosynthesis (Figure 1), and the transcriptional regulator FapR (*B. subtilis*) has been successfully used as a biosensor for dynamic control of FA biosynthesis in bacteria, reducing impaired cell growth caused by FA accumulation (Xu et al., 2014; Lo et al., 2016). FapR was also adapted to asses real time malonyl-CoA concentration in yeast (Dabirian et al., 2019) and mammalian cells (Ellis and Wolfgang, 2012), demonstrating the utility of these prokaryotic transcriptional regulators to function as metabolite sensors in eukaryotes. Additionally, when fused to nano-luciferase and targeted to subcellular compartments, FapR was shown to detect malonyl-CoA levels in mammalian cell organelles (Du et al., 2019). Malonyl-CoA is also a substrate for the first committed step in flavonoid biosynthesis, where chalcone synthase condenses three malonyl-CoA with *p*-coumaroyl-CoA to form naringenin chalcone. Even though, FapR has not been used in this context, dynamic regulation of malonyl-CoA concentration has been addressed to develop a naringenin producing *E. coli* strain (Zhou et al., 2021). With a multi-layered biosensor approach, these authors engineered a strain containing the genes for producing naringenin from tyrosine (layer I), combined with an FdeR-based module (layer II) that represses fatty acid biosynthesis in response to increased naringenin concentration. This repression allowed efficient redirection of malonyl-CoA to naringenin, while still maintaining fatty acid biosynthesis necessary for cell growth. Finally, a PadR-based module (layer III), responsive to *p*-coumaric acid (an intermediate of layer I), was included to enhance malonyl-CoA production *via* inhibition of acetyl-CoA consumption and overexpression of acetyl-CoA carboxylase. This strategy achieved dynamic control of malonyl-CoA consumption and allowed a balance between cell growth and naringenin production. Hence, although never used as a flavonoid-related biosensor, the importance of malonyl-CoA for flavonoid biosynthesis and the successful application of FapR in other eukaryotic cells suggest that FapR can be used as a key transcriptional regulator to obtain dynamic control of carbon flux into the flavonoid branch in plants.

## Genetic Circuit Input From Biosensors

Biosensors that respond to endogenous plant metabolites can also be used to provide inputs for synthetic genetic circuits that perform more complex operations in the cell. Genetic circuits are assemblies of biological components that perform logical functions or operations in the cell based on interactions among the various circuit components. Their operation is modulated by the presence of one or more specific inputs, followed by information processing according to pre-defined instructions, resulting in production of an output, most commonly a transcriptional response. Binding of ligands to sensor proteins and initiation of transcriptional responses can serve as inputs for these genetic circuits. Plant metabolite biosensors might function as the interface between the introduced genetic circuit and the plant’s natural metabolism, supplying real-time information on the concentration of key metabolites in the cell (Figures 2C,D). Complex biological computations triggered by changes in the levels of multiple endogenous plant metabolites (i.e., metabolic status) could then allow the development of more efficient strategies for control of metabolism in plants.

A hallmark of genetic circuits is the functional interaction among components, for example, in the form of feedback or feed-forward controls, and logic operations. Protein–protein and protein-DNA interactions are most common in these genetic circuits, with these interactions typically exerting transcriptional control over one or multiple downstream genes (Slusarczyk et al., 2012; Xia et al., 2019). RNA-based genetic circuits have also been explored (Kushwaha et al., 2016; Chappell et al., 2017; Nshogozabahizi et al., 2019), potentially allowing faster and more tunable circuit responses. Because of the various interactions, quantitative characterization of the behavior of each circuit component is essential for the development of accurate mathematical models, which aid both in the selection of optimal components and in the ability to predict circuit function (Brophy and Voigt, 2014). Biosensors are characterized by their dose–response curves, or transfer functions, which provide quantitative measurements of biosensor output relative to the amount of input. For plants, previous work has demonstrated that this characterization process may be achieved by transient expression of components in protoplasts (Schaumberg et al., 2016) or by leaf agro-infiltration (Bernabé-Orts et al., 2020), with significant time savings compared to stable transformation of plants.

Designing synthetic genetic circuits based on phenylpropanoid biosensors that trigger changes in gene expression to alter carbon flux through pathway branches is an exciting opportunity toward achieving dynamic control of specialized metabolites and designing plant biofactories.

## Future Perspectives

Plant phenylpropanoid metabolism has considerable importance to humans. The various products synthesized *via* its many branches have been exploited mainly for their health-related properties (Neelam et al., 2020). Therefore, efforts to engineer this pathway for improved efficiency and yield have received much attention, both in plants and with heterologous expression of enzymes in other hosts. Over-expression of transgenes may result in an unnecessary metabolic burden to the organism, especially if it involves many enzymes of a pathway, due to the energetic demand of continuous transcriptional and translational processes, as well as by the constitutive deviation from pathway “wild-type flux.” Hence, implementation of appropriate genetic controls to restrict transgene expression to meet demand only in limited tissues, developmental stages, energetic status, or stress conditions, will allow more efficient and less intrusive metabolic engineering approaches to be developed.

Synthetic biology offers the prospect of more advanced methods to design these “on-demand” genetic circuits, which must be able to sense the organism’s metabolic status to produce adequate activation of a specific pathway. In this context, expansion of the repertoire of sensing proteins – adapted from natural proteins or developed anew – will play a fundamental role. Adaptation of naturally occurring proteins to recognize new metabolites (ligands) may be achieved *via* structure-based rational modifications (Kuhlman and Bradley, 2019) or directed evolution (Chowdhury and Maranas, 2020) of their ligand-binding regions. Alternatively, new sensor proteins to metabolic ligands of interest may soon be designed entirely *de novo* using newer protein engineering algorithms (Lucas and Kortemme, 2020; Quijano-Rubio et al., 2021). Biosensors will thus be key to engineering dynamic control of not only phenylpropanoid metabolism, but also other primary and secondary metabolic plant pathways.

## Author Contributions

Both authors conceptualized, wrote and edited the manuscript. Both authors approved the submitted version.

## Funding

This research was funded with start-up funds to MA provided by the University of North Texas.

## Conflict of Interest

The authors declare that the research was conducted in the absence of any commercial or financial relationships that could be construed as a potential conflict of interest.

## Publisher’s Note

All claims expressed in this article are solely those of the authors and do not necessarily represent those of their affiliated organizations, or those of the publisher, the editors and the reviewers. Any product that may be evaluated in this article, or claim that may be made by its manufacturer, is not guaranteed or endorsed by the publisher.
